# Complete Mitochondrial Genome of *Pseudocaranx dentex* (Carangidae, Perciformes) Provides Insight into Phylogenetic and Evolutionary Relationship among Carangidae Family

**DOI:** 10.3390/genes12081234

**Published:** 2021-08-11

**Authors:** Busu Li, Huan Wang, Long Yang, Shufang Liu, Zhimeng Zhuang

**Affiliations:** 1 Yellow Sea Fisheries Research Institute, Chinese Academy of Fishery Sciences, Qingdao 266071, China; bsli@qnlm.ac (B.L.); wanghuan@ysfri.ac.cn (H.W.); yanglong_001@126.com (L.Y.); zhuangzm@ysfri.ac.cn (Z.Z.); 2 Laboratory for Marine Fisheries Science and Food Production Processes, Pilot National Laboratory for Marine Science and Technology (Qingdao), Qingdao 266237, China; 3 College of Fisheries, Zhejiang Ocean University, Zhoushan 316022, China

**Keywords:** *Pseudocaranx dentex*, mitogenome, phylogeny, evolution, Carangidae

## Abstract

*Pseudocaranx dentex* (white trevally) which belongs to the Carangidae family, is an important commercial fishery and aquaculture resource in Asia. However, its evolution and population genetics have received little attention which was limited by the mitogenome information absence. Here, we sequenced and annotated the complete mitochondrial genome of *P. dentex* which was 16,569 bp in length, containing twenty-two tRNAs (transfer RNAs), thirteen PCGs (protein-coding genes), two rRNAs (ribosomal RNAs), and one non-coding region with conservative gene arrangement. The Ka/Ks ratio analysis among Carangidae fishes indicated the PCGs were suffering purify selection and the values were related to the taxonomic status and further influenced by their living habits. Phylogenetic analysis based on the PCGs sequences of mitogenomes among 36 species presented three major clades in Carangidae. According to the phylogenetic tree, we further analyzed the taxonomic confusion of *Carangoides equula* which was on the same branch with *P. dentex* but a different branch with *Carangoides* spp. We inferred *Kaiwarinus equula* should be the accepted name and belong to the independent *Kaiwarinus* genus which was the sister genus of *Pseudocaranx*. This work provides mitochondrial genetic information and verifies the taxonomic status of *P. dentex*, and further helps to recognize the phylogenetic relationship and evolutionary history of Carangidae.

## 1. Introduction

Mitochondria, the main place that accounts for the energy support of various life activities [1], contains their own store of genetic information, which is the mitochondrial genome. The mitochondrial genome is typically circular in teleost which contains 13 protein-coding genes (PCGs), 22 transfer RNAs, 2 ribosomal RNAs, and one non-coding sequence. The mitochondrial species-specific DNA fragments, such as cytochrome c oxidase I (*COI*), cytochrome b (*CYTB*), and ribosomal RNA (12S and 16S) are molecular markers usually used for species identification and phylogenetic analysis in fishes [2,3,4]. However, single genes are too short to accurately reflect the real phylogenetic relationships among species and distinguish certain congeneric species. In contrast, the mitochondrial genome which contains greater sequence and additional informative sites has been regarded as a powerful molecular marker to identify phylogenetic relationships and explore population genetics and taxonomic diagnosis because of its high conservation, maternal inheritance, compact gene arrangement, and easy detection [5].

The Carangidae family belongs to Perciformes, suborder Percoidei, comprises 30 genera and approximately 147 species worldwide [6], and it is a diverse and economically important group distributed in all tropical, sub-tropical, and temperature marine water of the world [7,8]. Four subfamilies were recognized following Gushiken [9] published the phylogenetic tree. The properties of Carangidae’s meat, which is tasty with a high nutritional value, make them highly valuable for consumption, which further positions it as an essential pelagic resource in capture fisheries around the world. According to FAO (Fisheries and Aquaculture Department of Food and Agriculture Organization) statistics, the annual global capture production quantity of Carangidae fishes reached 5.13 million tons in 2018 (http://www.fao.org/fishery/statistics/, accessed on 25 March 2021), of which Asia accounted for 65%. Among Carangidae fishes, the aquaculture production of white trevally (*P. dentex*) (Bloch and Schneider, 1801) is the fourth largest in 2018, based on the FAO statistical data. *P. dentex* is a commercially important shallow reef fish that occurs on continental and island shelves across the anti-tropical regions of the Indo-Pacific, the Atlantic, and the Mediterranean [10]. Previous studies have examined the learning behavior [11] and physiological features [12] of *P. dentex*, but not its genetic characteristics and evolutionary patterns which are limited by the absence of mitogenome information. Furthermore, the nomenclature and taxonomy of some Carangidae fishes are disputed. As an example of the taxonomic confusion in the family, Gushiken considered *Kaiwarinus* as a sister genus to *Pseudocaranx* [9], whereas Eschmeyer listed *Kaiwarinus* as a synonym of *Carangoides* [13]. From the above, the mitogenome character identification of *P. dentex* is the foundation of population genetics research of *P. dentex* and will further help to recognize the evolutionary relationships of the Carangidae family and provide evidence for taxonomic classification of controversial species.

In the present study, we sequenced and annotated the complete mitochondrial genome of *P. dentex*. The genome composition and characteristics were described and the relative synonymous codon usage (RSCU) and AT-skew values of the PCGs were calculated. Furthermore, we analyzed phylogenetic relationships, taxonomic status, and selective pressures among the Carangidae species. The mitochondrial genetic information of *P. dentex* will help to enhance the limited molecular data available for the Carangidae species and further recognize the phylogenetic relationship of Carangidae.

## 2. Materials and Methods

### 2.1. Sample Collection and DNA Extraction

One *P. dentex* was collected from Dalian Tianzheng Industrial Co., Ltd. (Dalian, China). One-year *P. dentex* dorsal muscles were sampled and stored in anhydrous alcohol for subsequent DNA extraction. Genomic DNA was isolated from muscles using the TIANamp Marine Animals DNA Kit (DP324, Tiangen, Beijing, China), following the manufacturer’s protocol. DNA integrity and quality were assessed by 1% agarose gel electrophoresis, and their concentration was detected by a Nanodrop spectrophotometer. All experiments were conducted with approval by the Institutional Animal Care and Use Committee (IACUC) of the Yellow Sea Fisheries Research Institute (YSFRI), Chinese Academy of Fishery Sciences.

### 2.2. Library Construction and DNA Sequencing

High-quality DNA was randomly fragmented to about 350 bp. After terminal repair, adapters were ligated to both ends of the fragment. The libraries were generated for sequencing in the Illumina Hiseq2500 instrument (Illumina, San Diego, CA, USA), followed by amplification, size selection, and purification. Library construction and sequencing were performed by the Novogene Corporation (Beijing, China).

### 2.3. Sequence Assembly, Annotation, and Analysis

MitoZ [14] was used for the complete mitochondrial genome de novo assembly using the Kmer mode. The mitogenome was annotated with the MITOS Web server [15] using the vertebrate mitochondrial genetic code. The complete mitogenomes of *P. dentex* were uploaded to GenBank with the accession number MZ359280. The circular mitogenome map was visualized using the Circular Genome View Server [16]. Base composition and amino acid distributions were calculated with MEGA X [17]. The mitochondrial strand asymmetry was measured using the following formulas: GC-skew = (G − C)/(G + C) and AT-skew = (A − T)/(A + T). Codon usage was displayed as the codon frequency, and relative synonymous codon usage (RSCU) values were determined using CodonW.

### 2.4. Molecular Phylogenetic Analysis

The phylogenetic status of *P. dentex* in the family Carangidae was assessed by comparing concatenated sequences of 13 PCGs (without stop codon) of 36 Carangidae species across 18 genera, including the *P. dentex* mitogenome generated from the present study and others downloaded from NCBI (Table 1). *Larimichthys crocea* (Richardson, 1846) was chosen as an outgroup. We performed sequence alignment using the MAFFT program on the EMBL-EBI analysis tool [18] with default parameters chosen according to the instructions. After deleting total gaps, the automatic model test and Maximum likelihood (ML) tree construction were performed by IQtree [19]. The optimal evolution model was GTR + I + G based on the AIC (Akaike Information Criterion) in IQtree model analysis. The ML analysis was run with 1000 bootstrap replicates by IQtree. The final trees were visualized by Figtree.

### 2.5. Ka/Ks Analysis

The selective pressure magnitude and direction were displayed as the ratio of nonsynonymous substitutions number per nonsynonymous site (Ka) to the synonymous substitutions number per synonymous site (Ks), which was represented as w (w = Ka/K_S_). The positive, neutral, and purify selection corresponded to w values >1, =1, and <1, respectively [20]. The Ka/Ks ratio values of the Carangidae species were calculated in DNAsp [21] with mitochondrial PCGs after alignment with ClustalW.

## 3. Results

### 3.1. General Characteristics of the Pseudocaranx Dentex Mitogenome

**Figure 1:**
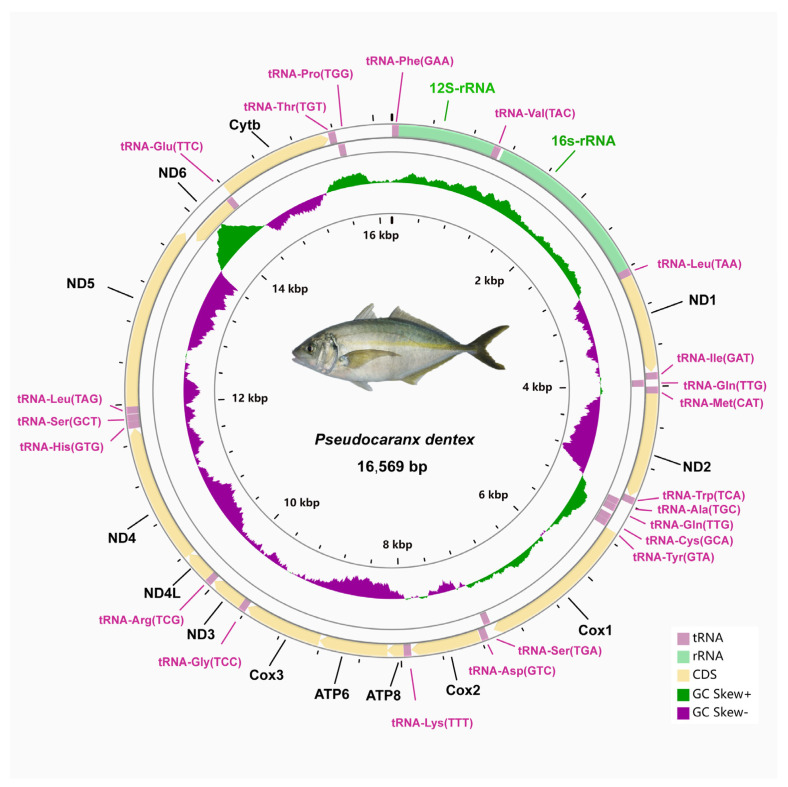
Circular mitochondrial genome map of *P. dentex*. The larger circle indicated the gene arrangement and distribution. Genes encoded by the H-strand and L-strand were displayed in outside and inner rings, respectively. The smaller circle indicated GC- and AT-skew distribution.

The circular mitogenome of *P. dentex* was 16,569 bp in length consists of thirteen protein-coding genes (PCGs), twenty-two transfer RNAs, two ribosomal RNAs, and one non-coding region (Figure 1 and Table 2). The total length of the PCGs was 11,430 bp, which accounts for 68.98% of the entire mitogenome. The 16 s rRNA and 12s rRNA genes were 1689 bp and 951 bp in length, respectively. The AT- and GC-skew of rRNAs were 0.1801 and −0.0897, respectively (Table 3). The total length of 22 tRNAs was 1556 bp with 0.0455 GC skewness and 0.0211 AT skewness. Their size ranged from 67 bp (tRNA-*Cys*) to 75 bp (tRNA-*Lys*).

Among these 37 genes, eight tRNA genes (trnQ, trnA, trnN, trnC, trnY, trnS2, trnE, trnP) and the ND6 gene were located in the light strand and the others were encoded by heavy strand (Figure 1 and Table 2). The structure and arrangement were in good agreement with other Carangidae fishes [22]. The base composition of the complete mitogenomes was listed as follows: A% = 25.40, T% = 27.21, C% = 17.18, G% = 30.21 (Table 3). The proportion of A+T (52.61%) was slightly higher than that of G+C (47.39%). The gene with the highest GC content was identified in *ND4* (53.16%). Consistent with other Carangidae fishes, the *P. dentex* mitogenome had a positive GC-skew (0.02751) and negative AT-skew (−0.0343) (Table 3).

### 3.2. PCGs and Codon Usages

The nucleotide composition of the PCGs was: T% (27.07%), C% (30.99%), A% (24.74%), and G% (17.20%), with an AT content of 51.81%. ATG was the most frequently used start codon in 12 PCGs, except for *COX1* initiating with codon GTG. TAA and TAG were the most frequent stop codon, while *COX2*, *ND4*, and *CYTB* genes had incomplete stop codon T. The AT-skew and GC-skew in PCGs of *P. dentex* were both negative, −0.0450 and −0.2859, respectively.

**Figure 2:**
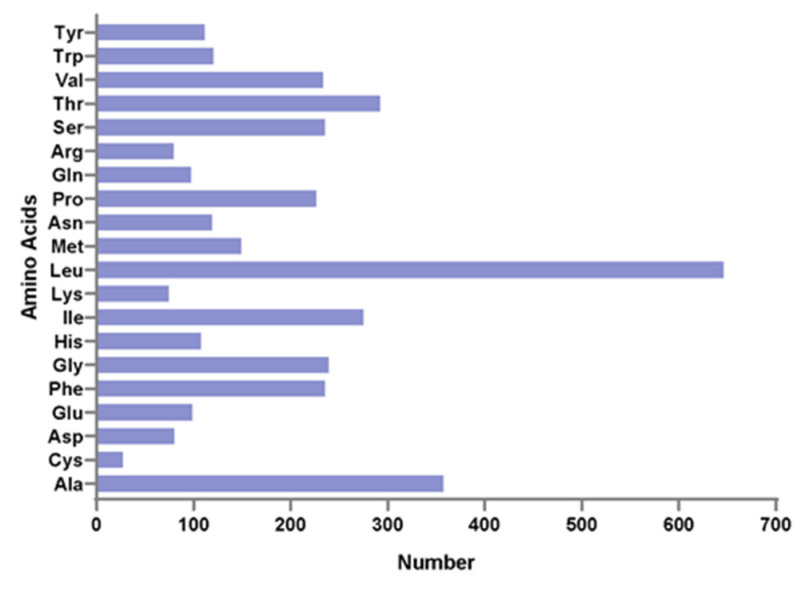
Amino acid distribution of the *P. dentex* mitochondrial genome. The horizontal axis and vertical axis are the number and types of amino acids, respectively.

**Figure 3:**
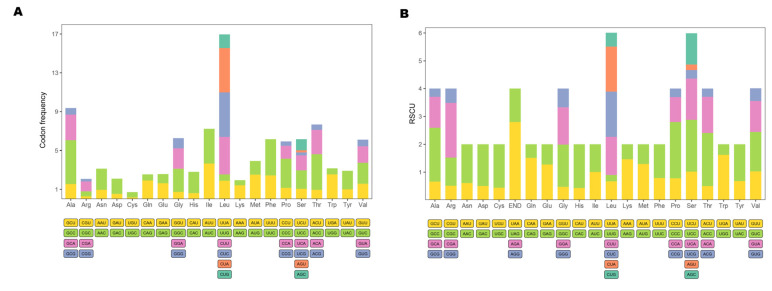
Codon usage frequency (**A**) and relative synonymous codon usage (**B**) in the *P. dentex* mitogenome. Amino acids are labelled on the x-axis and the codon families are displayed bellow.

The 13 protein-coding genes have 3809 codons. Leu, Ala, Thr, and Ile were the most abundant amino acids in mitogenome PCGs (Figure 2). Codon frequencies were calculated by the occurrence frequency of codons in all PCGs. Leu (CUC, CUA, CUU), Ala (GCC), Phe (UUC), and Thr (ACC) were the most abundant codons, while stop codon (UAG) and Cys (UGU) were the least frequent (Figure 3A). The RSCU is a widely used measurement for each codon usage bias of each amino acid. The RSCU value for a codon close to 1 means that there is no preference for that codon, and an RSCU value >1 indicates that there is a strong bias for that codon [23]. The RSCU values of the *P. dentex* genome are displayed in Figure 3B. Pro (CCC), Arg (CGA), Ala (GCC), Thr (ACC), Ser (UCC), and Leu (CUC and CUA) were the most frequently used codons, while Ser (UCG and AGU), Pro (CCG), Ala (GCG), Thr (ACG), Leu (UUG) were the least.

### 3.3. Ka/Ks Analysis

**Figure 4:**
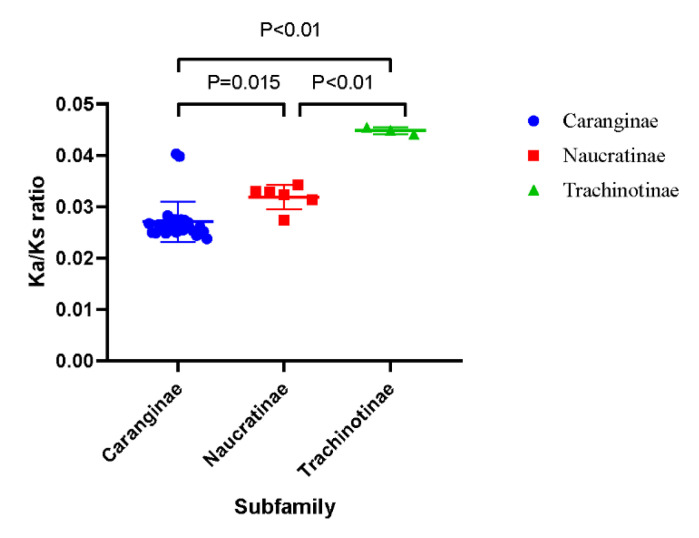
Plot of the individual Ka/Ks ratio values of three subfamilies.

The Ka/Ks ratio values were calculated to describe the selective pressure magnitude and direction of the PCGs. The Ks, Ka, and Ka/Ks ratio values of the PCGs are displayed in Table 4. The average Ka/Ks values of all Carangidae fishes were <1, which suggested the purifying selection of functional genes. Among these species, the Ka/Ks ratio values of the Trachinotiae subfamily were significantly larger than the Caranginae and Naucratinae subfamilies (Figure 4).

### 3.4. Phylogenetic Analysis

**Figure 5:**
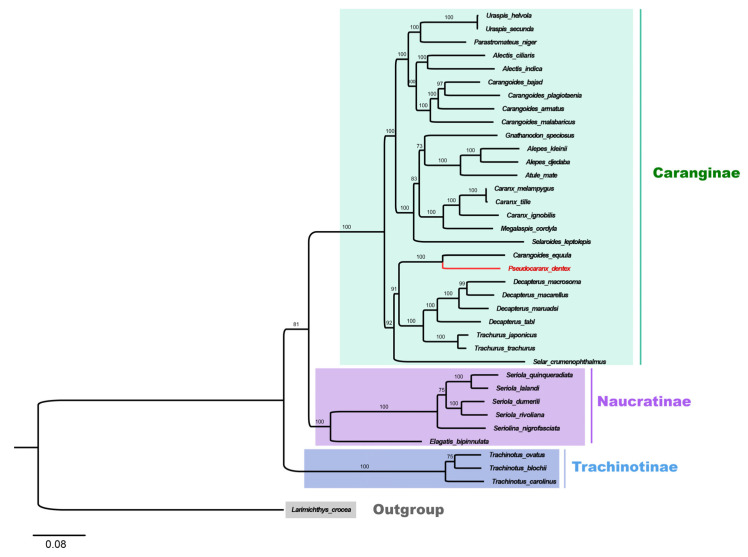
Phylogenetic tree of 36 species from the Carangidae family constructed by the maximum Likelihood method based on the concatenated sequences of 13 PCGs. *L. crocea* was set as an outgroup. The bootstrap support values were displayed on the branches. The species in red (*P. dentex*) was generated in the present study. Trachinotiae, Naucratinae, and Caranginae subfamilies were labeled in blue, purple, and green, respectively.

Based on the concatenated sequences of the mitochondrial PCGs, the phylogenetic relationship among 36 Carangidae fishes was analyzed. According to the phylogenetic tree, 36 Carangidae fishes showed a clear separation into three subfamilies: Trachinotiae, Naucratinae, and Caranginae. The Caranginae and Naucratinae subfamilies were clustered together and then clustered with the Trachinotiae subfamily (Figure 5). *P. dentex* is a species of the Caranginae subfamily.

**Figure 6:**
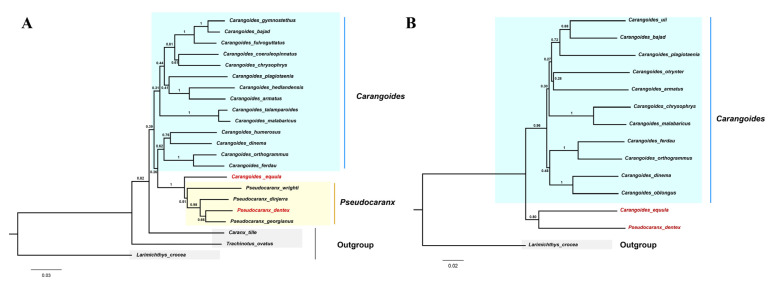
Neighbor-Joining trees based on *COI* (**A**) and *CYTB* (**B**) sequences from the Carangoides and Pseudocaranx genera together with *C. equula*. The numbers at the nodes were the bootstrap support values. *P. dentex* and *C. equula* were labeled in red. (**A**) *C. tille*, *T. ovatus,* and *L. crocea COI* sequences were set as outgroups. (**B**). *L. crocea* was an outgroup.

In the Caranginae subfamily, the naming and classification of *Carangoides equula* remains controversial. *C. equula* was once thought to belong to an independent *Kaiwarinus* genus, whereas, in the present taxonomic system of FishBase and WoRMS, *Kaiwarinus equula* is considered a synonym of *Carangoides equula* [9,24,25]. According to our phylogenetic tree, based on the mitochondrial PCGs sequences in 36 Carangidae fishes, *C. equula* was on the same branch with *P. dentex* but on a different branch with the *Carangoides* spp. Considering only a few species of mitochondrial genome information were available in the *Pseudocaranx* and *Carangoides* genus, we constructed Neighbor-Joining phylogenetic trees with *COI* and *CYTB* sequences of *Carangoides* and *Pseudocaranx* genera together with *C. equula* to further analyze its classification status. Eighteen *COI* sequences (14 in *Carangoides* and 4 in *Pseudocaranx*) and thirteen *CYTB* sequences (12 in *Carangoides* and 1 in *Pseudocaranx*) were downloaded from GenBank or extracted from mitochondrial genome sequences to construct the NJ phylogenetic trees. The sequence information of *COI* and *CYTB* are shown in Tables S1 and S2, respectively. Similar to the phylogenetic relation analysis based on the mitochondrial PCGs sequences, *Carangoides equula* was on a different branch with the *Carangoides* spp., but in the same subgroup with the *Pseudocaranx* spp. (Figure 6A,B). According to the above three phylogenetic trees, we inferred *Kaiwarinus equula* was the accepted name and should belong to the independent *Kaiwarinus* genus which is the sister genus of *Pseudocaranx*.

## 4. Discussion

**Figure 7:**
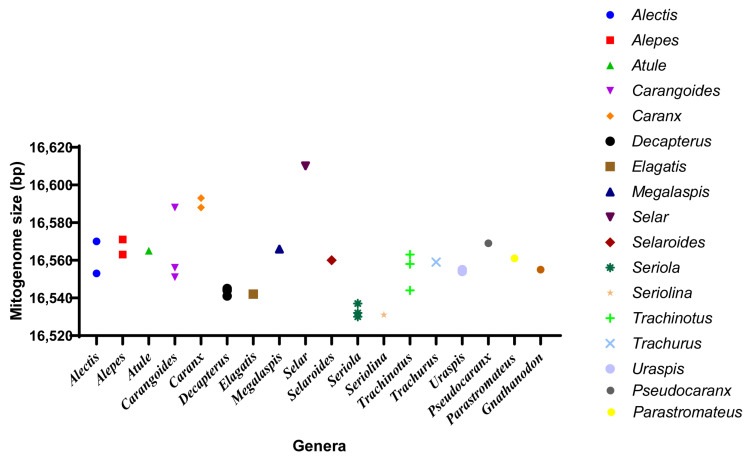
Mitogenome size distribution of Carangidae fishes. Thirty-six species from eighteen genera obtained from GenBank were displayed.

We sequenced the complete mitochondrial genome of *P. dentex* and summarized the mitogenome information of 36 species in 19 genera of the Carangidae family. The total mitochondrial genome length of the Carangidae family ranged from 16,530 bp (*S. dumerili*) [26] to 16,610 bp (*S.crumenophthalmus*) [27] with minor differences (Figure 7 and Table 1), wherein, the length of *P. dentex* is 16,569 bp. The mitogenomic structure of *P. dentex* was conserved in the Carangidae family without gene rearrangement, which is consistent with most vertebrates.

### 4.1. Mitochondrial Genetic Code Differed from Nuclear Universal Code

The mitochondrial genetic codes differ from nuclear universal codes; also, the mitochondrial codes are various among vertebrates, yeast, invertebrates, algae, bacteria, fungi, and metazoan [28]. In *P. dentex*, most genes were initiated by the ATG codon and terminated with TAA and TAG codons, while *COX1* had the GTG start codon and *COX2*, *ND4*, *CYTB* genes had incomplete stop codons, T--. The incomplete termination codon was a common genetic codon in the mitochondrial genome. The model to explain the incomplete stop codons was that the addition of a Poly(A) to the 3′-terminal of reading frames with U-- or UA- could create the UAA stop codons post-transcriptionally [29,30].

### 4.2. Usage of Mitogenome Codon

Leu, Ala, Thr, and Ile were the most abundant amino acids in mitogenome PCGs, which were also the most frequent amino acids in other teleost mitogenomes [22,31,32]. The RSCU is a parameter to assess the synonymous codon usage bias. Pro (CCC), Arg (CGA), Ala (GCC), Thr (ACC), Ser (UCC), and Leu (CUC and CUA) were the most frequently used codons, while Ser (UCG and AGU), Pro (CCG), Ala (GCG), Thr (ACG), and Leu (UUG) were the least in the *P. dentex* mitochondrial genome, which was not exactly the same as other Carangidae species [22]. In multiple species, codon usage was related to the gene expression level, gene length, tissues [33], and even influenced protein sequences [34]. Differences in codon usage frequency in Carangidae might be related to natural (translation) selection [35] and mutation pressure processes [33] among various species.

### 4.3. Selective Pressure in Carangidae Family

The Ka/Ks ratio of mitochondrial PCGs is the representative parameter of selective pressure magnitude and direction. There is growing evidence that the accumulation of mtDNA mutation is influenced by life history, effective population size, and even cellular energy requirements [36]. It has been found that species in high-performance groups displayed lower Ka/Ks ratios compared to weakly locomotive species in birds [37], mammals [38], and teleost [39]. Among the Carangidae family, the Ka/Ks ratio of PCGs in Trachinotiae was significantly higher than the other two subfamilies. In these three subfamilies, most species of Caranginae and Naucratinae exhibited migratory behavior with high performance [7,40]. For instance, *P. dentex* in the present study, which belongs to the Caranginae subfamily, was a highly vagile fish species and performed seasonal migration [41]. While *T. carolinus*, *T. ovatus*, and *T. blochii* were reported to occur in coastal or offshore environments [42] with weaker swimming ability and limited living range relative to the other two subfamilies, leading to a small effective population subjected to weaker purification selection, which further accumulated more nonsynonymous mutations in the Trachinotinae subfamily [36]. The Caranginae and Naucratinae subfamilies with higher performance showed a lower Ka/Ks ratio compared with the weakly locomotive Trachinotiae subfamily, which suggested that the Ka/Ks ratio of Carangidae fishes may be related to their taxonomic status, and further influenced by their living habits and swimming abilities.

### 4.4. Molecular Phylogeny of Carangidae Fishes

The Mitochondrial genome has become a powerful molecular marker for genetic analysis which can provide additional genetic informative sites and has high conservation and simple structural features [43,44]. Together with the sequences of *P. dentex* obtained in the present study, the mitochondrial genomes of a total of 36 Carangidae fishes were available. These 36 Carangidae fishes were from 18 genera and 3 subfamilies, except for the Scomberoidinae subfamily—the complete genome of which was absent. The dendrogram of the Carangidae family was drawn according to the concatenated mitochondrial PCGs sequences of 36 Carangidae fishes. Our phylogenetic analysis showed a clear separation of these 36 Carangidae fishes into three subgroups. Compared with a previous phylogenetic study based on mitochondrial genome information [22], we discussed phylogenetic relationships of five more species that belong to three genera in the Carangidae family. Our results supported Gushiken’s phylogenetic relationship analysis of 46 species in 32 genera of the Carangidae family, which was based on twenty-five morphological and osteological characters in 1988 [9]. Among Carangidae fishes, the naming and classification of *C. equula,* which belong to the Caranginae subfamily, still remains controversial [9,24,25]. It was once thought that *C. equula* should belong to the *Kaiwarinus* genus which was an independent genus and in the same branch as the *Pseudocaranx* genus based on morphological and osteological characteristics [9]. While, in the current taxonomic system of FishBase (https://fishbase.se/search.php, accessed on 8 June 2021) and WoRMS (https://www.marinespecies.org/, accessed on 8 June 2021), *Kaiwarinus equula* (Temminck and Schlegel, 1844) is listed as a synonym of *Carangoides equula* (Temminck and Schlegel, 1844). The generation of the *P. dentex* mitogenome in the present study was helpful to clarify the nomenclature and taxonomy of *C. equula*. By applying the whole mitochondrial genome, *COI*, and *CYTB* genes of available Carangidae fishes, our results confirmed *C. equula* should belong to the *Kaiwarinus* genus, in line with results from other scholars [9,12,45,46]. Combining the results discussed above, we inferred that *Kaiwarinus equula* should be the accepted name instead of *Carangoides equula* and belong to an independent genus which is the sister genus of *Pseudocaranx*.

## 5. Conclusions

In summary, we sequenced and annotated the complete mitochondrial genome of *P. dentex* which was 16,569 bp with conservative gene arrangement. The evolutionary relationship analysis revealed that the Trachinotiae subfamily showed a significantly higher Ka/Ks ratio relative to the Naucratinae and Caranginae subfamilies, which indicated that the Ka/Ks ratio values were correlated with the taxonomic status and their living habits. Phylogenetic analysis provided a further supplement to the scientific classification of Carangidae fishes. Based on the phylogenetic tree topology, we inferred *Kaiwarinus equula* was the accepted name of *Carangoides equula* and belongs to an independent *Kaiwarinus* genus which is the sister genus of *Pseudocaranx*. This work enriched mitochondrial genome information of the Carangidae family and will greatly improve our understanding of the evolution profile and phylogenetic position of the Carangidae family.

## Figures and Tables

**Table 1:** Taxonomic information and GenBank accession numbers of all species cited in the mitogenome PCGs phylogenetic analysis.

Subfamily	Genus	Species	Mitogenome Length (bp)	Accession Number
Caranginae	*Alectis*	*Alectis ciliaris*	16,570	KM522837
		*Alectis indica*	16,553	KP710215
	*Alepes*	*Alepes djedaba*	16,563	KP408222
		*Alepes kleinii*	16,571	KF728081
	*Atule*	*Atule mate*	16,565	KM522838
	*Carangoides*	*Carangoides armatus*	16,556	AP004444
		*Carangoides malabaricus*	16,561	KJ174514
		*Carangoides plagiotaenia*	16,551	MT677872.1
		*Carangoides bajad*	16,556	LC557137
		*C.equula*	16,588	KM201334
	*Caranx*	*Caranx ignobilis*	16,588	KF649842
		*Caranx melampygus*	16,593	AP004445
		*Caranx tille*	16,593	NC_029421.1
	*Decapterus*	*Decapterus macarellus*	16,544	KM986880
		*Decapterus macrosoma*	16,545	KF841444
		*Decapterus maruadsi*	16,541	KJ004518
		*Decapterus tabl*	16,545	MN102718
	*Megalaspis*	*Megalaspis cordyla*	16,566	KM522836
	*Selar*	*Selar crumenophthalmus*	16,610	KJ148633
	*Trachurus*	*Trachurus japonicus*	16,559	AP003091
		*Trachurus trachurus*	16,559	AB108498
	*Uraspis*	*Uraspis helvola*	16,555	KM978993
		*Uraspis secunda*	16,554	KT819204
	*Parastromateus*	*Parastromateus niger*	16,561	KJ192332
	*Pseudocaranx*	*P. dentex*	16,569	Present study
	*Gnathanodon*	*Gnathanodon speciosus*	16,555	MT922005
	*Selaroides*	*Selaroides leptolepis*	16,560	KM522839
Naucratinae	*Seriola*	*Seriola dumerili*	16,530	AB517558
		*Seriola lalandi*	16,532	AB517557
		*Seriola quinqueradiata*	16,537	AB517556
		*Seriola rivoliana*	16,530	KP347126
	*Seriolina*	*Seriolina nigrofasciata*	16,531	KT591876
	*Elagatis*	*Elagatis bipinnulata*	16,542	KT824759
Trachinotiae	*Trachinotus*	*Trachinotus blochii*	16,558	KJ184305
		*Trachinotus carolinus*	16,544	KJ556976
		*Trachinotus ovatus*	16,563	KF356397
Outgroup	*Larimichthys*	*L.crocea*	16,466	NC_011710.1

**Table 2:** Genome features of *P. dentex*.

Gene	Strand	Location	Size (bp)	Intergenics Length	Anticodon	Amino Acids	Start Codon	Stop Codon
*tRNA^Phe^*	+	1-68	68	0	GAA			
*12s-rRNA*	+	69-1019	951	0	-			
*tRNA^Val^*	+	1020-1091	72	29	TAC			
*16s-rRNA*	+	1121-2809	1689	0	-			
*tRNA^Leu^*	+	2810-2883	74	0	TAA			
*ND1*	+	2884-3858	975	5	-	324	ATG	TAA
*tRNA^Ile^*	+	3864-3933	70	−1	GAT			
*tRNA^Gln^*	−	3933-4003	71	−1	TTG			
*tRNA^Met^*	+	4003-4071	69	0	CAT			
*ND2*	+	4072-5118	1047	−2	-	348	ATG	TAG
*tRNA^Trp^*	+	5117-5187	71	1	TCA			
*tRNA^Ala^*	−	5189-5257	69	1	TGC			
*tRNA^Gln^*	−	5259-5331	73	2	GTT			
*tRNA^Cys^*	−	5368-5434	67	0	GCA			
*tRNA^Tyr^*	−	5435-5504	70	1	GTA			
*COX1*	+	5506-7056	1551	0	-	516	GTG	TAA
*tRNA^Ser^*	−	7057-7127	71	3	TGA			
*tRNA^Asp^*	+	7131-7201	71	7	GTC			
*COX2*	+	7209-7899	691	0	-	230	ATG	T--
*tRNA^Lys^*	+	7900-7974	75	1	TTT			
*ATP8*	+	7976-8140	165	−7	-	54	ATG	TAG
*ATP6*	+	8134-8817	684	−1	-	227	ATG	TAA
*Cox3*	+	8817-9602	786	−1	-	261	ATG	TAA
*tRNA^Gly^*	+	9602-9671	70	0	TCC			
*ND3*	+	9672-10022	351	−2	-	116	ATG	TAG
*tRNA^Arg^*	+	10021-10089	69	1	TCG			
*ND4L*	+	10091-10387	297	−7	-	98	ATG	TAA
*ND4*	+	10381-11761	1381	0	-	460	ATG	T--
*tRNA^His^*	+	11762-11833	72	0	GTG	23		
*tRNA^Ser^*	+	11834-11901	68	4	GCT			
*tRNA^Leu^*	+	11906-11978	73	0	TAG			
*ND5*	+	11979-13817	1839	−4	-	612	ATG	TAA
*ND6*	−	13814-14335	522	0	-	173	ATG	TAA
*tRNA^Glu^*	−	14336-14404	69	4	TTC			
*Cytb*	+	14409-15549	1141	0	-	380	ATG	T--
*tRNA^Thr^*	+	15550-15622	73	−1	TGT			
*tRNA^Pro^*	−	15622-15692	71	299	TGG			
D-Loop	+	15992-16508	517	61	-			

**Table 3:** Nucleotide composition and strand asymmetry of the *P. dentex* mitochondrial genome.

	T%	C%	A%	G%	AT%	GC-Skew%	AT-Skew%
PCGs	27.0696	30.9855	24.7394	17.2054	51.8090	−0.2859	−0.0450
tRNA	26.7995	21.5938	27.9563	23.6504	54.7558	0.0455	0.0211
rRNA	21.8177	25.4879	31.4011	21.2933	53.2188	−0.0897	0.1801
Control region	28.4333	25.7253	28.6267	17.2147	57.0600	−0.1982	0.0034
Genome	27.2074	17.1767	25.4029	30.2130	52.6103	0.2751	−0.0343

**Table 4:** The mean values of the Ka/Ks ratio for the PCGs in the pairwise mitochondrial genome among the Carangidae fishes.

Subfamily	Species	Ks Average	Ka Average	Ka/Ks Ratio
Caranginae	*A. ciliaris*	1.11493	0.02872	0.02576
	*A. dica*	1.12443	0.03068	0.02729
	*A. djedaba*	1.16419	0.03107	0.02669
	*A. kleinii*	1.16015	0.03191	0.02751
	*A. mate*	1.23389	0.03272	0.02652
	*C. armatus*	1.09701	0.02795	0.02547
	*C. bajad*	0.99860	0.02605	0.02609
	*C. equula*	1.21870	0.04904	0.04024
	*C. malabaricus*	1.11391	0.02800	0.02513
	*C. plagiotaenia*	1.10618	0.02793	0.02525
	*C. ignobilis*	1.08045	0.02787	0.02580
	*C. melampygus*	1.00735	0.02728	0.02708
	*C. tille*	1.00936	0.02755	0.02730
	*D. macarellus*	1.13568	0.02764	0.02434
	*D. macrosoma*	1.15355	0.02885	0.02501
	*D. maruadsi*	1.10488	0.02741	0.02481
	*D. tabl*	1.12607	0.02673	0.02373
	*G. speciosus*	1.10255	0.03112	0.02823
	*M. cordyla*	1.09730	0.03009	0.02742
	*P. niger*	1.03399	0.02630	0.02543
	*P. dentex*	1.14894	0.04570	0.03978
	*S. crumenophthalmus*	1.27882	0.03437	0.02688
	*S. leptolepis*	1.23398	0.03213	0.02603
	*U. helvola*	1.07254	0.02674	0.02493
	*U. secunda*	1.06888	0.02656	0.02485
	*T. japonicus*	1.05488	0.02762	0.02619
	*T. trachurus*	1.04499	0.02763	0.02644
Naucratinae	*S. dumerili*	1.33503	0.04409	0.03302
	*S. lalandi*	1.36171	0.04401	0.03232
	*S. quinqueradiata*	1.38865	0.04350	0.03132
	*S. rivoliana*	1.30089	0.04452	0.03422
	*S. nigrofasciata*	1.38574	0.04552	0.03285
	*E. bipinnulata*	1.43412	0.03916	0.02731
Trachinotiae	*T. blochii*	1.39809	0.06355	0.04545
	*T. carolinus*	1.45539	0.06421	0.04412
	*T. ovatus*	1.42045	0.06373	0.04487

## Data Availability

The complete mitogenome of *P. dentex* was available in GenBank with accession number MZ359280.

## Supplementary Materials

The following are available online at https://www.mdpi.com/article/10.3390/genes12081234/s1, Table S1: Information of the *COI* gene sequences used in the phylogenetic analysis, Table S2. Information of the *Cytb* gene sequences used in the phylogenetic analysis.
