# Supplementary material for: Complete Mitochondrial Genome of Pseudocaranx dentex (Carangidae, Perciformes) Provides Insight into Phylogenetic and Evolutionary Relationship among Carangidae Family
Source: Genes (Basel). 2021 Aug 11;12(8):1234. doi: 10.3390/genes12081234 (PMC8392498; doi:10.3390/genes12081234)
Supplement: Supplementary file 1 [file genes-12-01234-s001.zip › genes-1310654-supplementary.pdf]

Table S1. Information of the *COI* gene sequences used in the phylogenetic analysis.

Genus	Species	Gene length(bp)	Accession number
Carangoides	*Carangoides talamparoides*	650	HQ560996.1
	Carangoides plagiotaenia	650	MT677872.1
	Carangoides orthogrammus	650	JQ431539.1
	Carangoides humerosus	650	HQ956543.1
	Carangoides hedlandensis	650	KC970455.1
	Carangoides gymnostethus	650	HQ560962.1
	Carangoides fulvoguttatus	650	EF609302.1
	Carangoides ferdau	650	KF714902.1
	Carangoides dinema	650	KX712509.1
	Carangoides coeruleopinnatus	650	KC970454.1
	Carangoides chrysophrys	650	KF009571.1
	Carangoides malabaricus	650	KJ174514
	Carangoides bajad	650	LC557137
	Carangoides armatus	650	AP004444
	Carangoides equula	650	KM201334
Pseudocaranx	*Pseudocaranx wrighti*	650	EF609443.1
	Pseudocaranx georgianus	650	MK101227.1
	Pseudocaranx dinjerra	650	MK101237.1
	Pseudocaranx dentex	650	Present study
**Outgroup**	*Trachinotus ovatus*	650	KF356397
**Outgroup**	*Caranx tille*	650	NC_029421.1
**Outgroup**	*Larimichthys crocea*	650	NC_011710.1

Table S2. Information of the *Cytb* gene sequences used in the phylogenetic analysis.

Genus	Species	Gene length(bp)	Accession number
Carangoides	*Carangoides otrynter*	1140	AF363741.1
	Carangoides chrysophrys	1140	AF363742.1
	Carangoides ferdau	1140	KF760447.1
	Carangoides orthogrammus	1140	KF760448.1
	Carangoides uii	1140	KF760449.1
	Carangoides dinema	1140	KF760450.1
	Carangoides oblongus	1140	KF760451.1
	Carangoides armatus	1140	AP004444
	Carangoides malabaricus	1140	KJ174514
	Carangoides plagiotaenia	1140	MT677872.1
	Carangoides bajad	1140	LC557137
	Carangoides equula	1140	KM201334
Pseudocaranx	*Pseudocaranx dentex*	1140	Present study
Outgroup	*Larimichthys crocea*	1140	NC_011710.1
